# Estimating peanut and soybean photosynthetic traits using leaf spectral reflectance and advance regression models

**DOI:** 10.1007/s00425-022-03867-6

**Published:** 2022-03-24

**Authors:** Ma. Luisa Buchaillot, David Soba, Tianchu Shu, Juan Liu, Iker Aranjuelo, José Luis Araus, G. Brett Runion, Stephen A. Prior, Shawn C. Kefauver, Alvaro Sanz-Saez

**Affiliations:** 1 Integrative Crop Ecophysiology Group, Plant Physiology Section, Faculty of Biology, University of Barcelona, 08028 Barcelona, Spain; 2 AGROTECNIO (Center for Research in Agrotechnology), Av. Rovira Roure 191, 25198 Lleida, Spain; 3 Instituto de Agrobiotecnología (IdAB), Consejo Superior de Investigaciones Científicas (CSIC)-Gobierno de Navarra, Av. Pamplona 123, 31192 Mutilva, Spain; 4 Department of Crop, Soil, and Environmental Sciences, Auburn University, Alabama, USA; 5 Industrial Crops Research Institute, Henan Academy of Agricultural Sciences, Henan, China; 6 U.S. Department of Agriculture–Agricultural Research Service, National Soil Dynamics Laboratory, Auburn, AL 36832 USA

**Keywords:** Advanced regression models, ARDR, Bayesian ridge model, High-throughput phenotyping, *J*_max_, Lasso, Leaf reflectance, Peanut, Photosynthesis, PLS, Soybean, *V*_*c*,max_

## Abstract

**Main conclusion:**

By combining hyperspectral signatures of peanut and soybean, we predicted *V*_cmax_ and *J*_max_ with 70 and 50% accuracy. The PLS was the model that better predicted these photosynthetic parameters.

**Abstract:**

One proposed key strategy for increasing potential crop stability and yield centers on exploitation of genotypic variability in photosynthetic capacity through precise high-throughput phenotyping techniques. Photosynthetic parameters, such as the maximum rate of Rubisco catalyzed carboxylation (*V*_*c*,max_) and maximum electron transport rate supporting RuBP regeneration (*J*_max_), have been identified as key targets for improvement. The primary techniques for measuring these physiological parameters are very time-consuming. However, these parameters could be estimated using rapid and non-destructive leaf spectroscopy techniques. This study compared four different advanced regression models (PLS, BR, ARDR, and LASSO) to estimate *V*_*c*,max_ and *J*_max_ based on leaf reflectance spectra measured with an ASD FieldSpec4. Two leguminous species were tested under different controlled environmental conditions: (1) peanut under different water regimes at normal atmospheric conditions and (2) soybean under high [CO_2_] and high night temperature. Model sensitivities were assessed for each crop and treatment separately and in combination to identify strengths and weaknesses of each modeling approach. Regardless of regression model, robust predictions were achieved for *V*_*c*,max_ (*R*^2^ = 0.70) and *J*_max_ (*R*^2^ = 0.50). Field spectroscopy shows promising results for estimating spatial and temporal variations in photosynthetic capacity based on leaf and canopy spectral properties.

**Supplementary Information:**

The online version contains supplementary material available at 10.1007/s00425-022-03867-6.

## Introduction

One of the great challenges for the future is the production of sufficient food for a growing population. From 1961 to 2012, the human population more than doubled from approximately 3 billion to 7 billion people and a further increase to 9.3 billion is projected for the year 2050 (FAOSTAT 2016). This means that crop production must double by 2050 to meet the predicted production demands of the global population. However, achieving this goal will be a significant challenge for agriculture since crop yields would have to increase at a rate of 2.4% per year, yet the average rate of increase is only 1.3%, with yields stagnating in up to 40% of land under cereal production (Araus and Cairns 2014). Further, climate change will exacerbate this challenge by intensifying field crop exposure to abiotic stress conditions, including rising temperature, drought, and increased CO_2_ concentration [CO_2_] (Christensen et al. 2007). This is a major issue because climatic factors since the end of the 1980s have counterbalanced the wheat genetic progress of recent decades in Europe (Oury et al. 2012). Indeed, as observed by Oury et al. (2012) and Gray and Brady (2016), the beneficial effects expected from the increase in atmospheric [CO_2_] in the World’s crop production during recent decades have been constrained by the effects of temperature increases and extended drought.

Grain legumes are the main source of proteins, minerals, and fibers for animals and humans (Meena et al. 2018). To achieve significant improvements in crop yield, breeding strategies aiming to increase biomass gains and crop productivity need to focus on radiation uptake, photosynthetic efficiency, and harvest index (HI) (Reynolds et al. 2012; Koester et al. 2014). However, to date, breeding for higher photosynthetic efficiency or for tolerance to different environmental stresses has only played a minor role in increasing crop productivity over past decades (Zhu et al. 2010). In a rational sense, plant physiology research should focus on improving photosynthesis due to its central part in plant productivity (Long et al. 2004). Recently, different studies have advanced how to optimize photosynthetic processes in different crops (Ort et al. 2015; Simkin et al. 2019).

One way to improve crop photosynthesis is to increase our knowledge of genomic control of photosynthesis under different environmental conditions. To achieve this, diverse crop populations representing hundreds of cultivars need to be screened (phenotyped) under different environments to associate traits of interest (i.e., photosynthetic parameters) with specific genomic regions. With the rise of genomic and bioinformatics technologies, phenotyping entire populations for traits of interest is the bottleneck that delays scientific advancement in genomics (Adachi et al. 2011; Yan et al. 2015; de Oliveira Silva et al. 2018; Oakley et al. 2018). Therefore, genomic approaches and breeding solutions need to implement new high-throughput phenotyping techniques that allow rapid measurement of photosynthetic traits for screening cultivars in the shortest amount of time (Araus and Cairns 2014; Araus et al. 2018). By improving techniques for measuring photosynthetic traits, more efficient cultivar selection will likely improve both yield potential and resilience to abiotic stresses.

Photosynthetic performance is frequently measured with an infrared gas analyzer that assesses plant CO_2_ assimilation rate. Photosynthetic parameters, such as leaf mid-day photosynthesis and leaf diurnal photosynthesis, can be used to assess in situ plant performance under different abiotic stresses (Sanz-Sáez et al. 2012, 2017). More detailed photosynthetic parameters, such as maximum rate of rubisco-catalyzed carboxylation (*V*_*c*,max_) and maximum electron transport rate supporting RuBP regeneration (*J*_max_), have been identified as selection parameters for tolerance to abiotic stress, such as drought (Aranjuelo et al. 2009, 2013), elevated tropospheric ozone (Yendrek et al. 2017), or for improved performance under elevated atmospheric CO_2_ (Ainsworth et al. 2004; Soba et al. 2020). Depending on the parameter to be measured, sampling can take a few minutes each (e.g., mid-day photosynthesis) or 20–60 min per sample for photosynthetic parameters, such as *V*_*c*,max_ and *J*_max_, which are calculated using photosynthesis to intercellular CO_2_ curves or A–Ci curves (Farquhar et al. 1980; Long and Bernacchi 2003). In addition, *V*_*c*,max_ and *J*_max_ are essential input parameters for the FvCB model (Farquhar et al. 1980) that relates photosynthetic biochemistry responses to known environmental conditions (Von Caemmerer 2013). This model has also been used in earth systems models for predicting ecosystem responses to environmental changes (Rogers 2014).

Reflectance spectra at leaf and canopy levels can facilitate assessment of plant’s structure, nutritional status, and certain stress parameters. This includes estimating contents of chlorophyll, xanthophylls, nitrogen, phosphorus, fiber, sucrose (Gamon et al. 1997; Peñuelas and Filella 1998; Petisco et al. 2006; Asner and Martin 2008; Colombo et al. 2008; Ainsworth et al. 2014; Serbin et al. 2014; Dechant et al. 2017; Yendrek et al. 2017), and plant secondary metabolites (Couture et al. 2016; Vergara-Diaz et al. 2020). In addition, leaf level spectral reflectance has been used to predict photosynthetic parameters, such as *V*_*c*,max_ and *J*_max_ in soybean (Ainsworth et al. 2014), wheat (Silva-Perez et al. 2018), maize (Heckmann et al. 2017; Yendrek et al. 2017), and trees (Serbin et al. 2012) as well as dark respiration in wheat (Coast et al. 2019).

Although translating data acquired with a field spectrometer using a leaf clip to scalable imaging approaches using multispectral or hyperspectral cameras in drones or other aerial platforms (frequently limited to the 350–1000 nm spectral range) may be further complicated by the heterogeneous nature of canopies, such techniques could greatly expand the scope of applicability of these measurements. In the above-mentioned research, relationships between photosynthetic parameters and complex data arrays captured by leaf level spectrometers need to be analyzed using complex multivariate statistical models. Partial least squares regression (PLSR) is the most commonly used model (Serbin et al. 2012; Ainsworth et al. 2014; Heckmann et al. 2017; Silva-Pérez et al. 2017; Yendrek et al. 2017). However, Fu et al. (2020) recently reported that other machine learning algorithms such as Least Absolute Shrinkage and Selection Operator (LASSO) can estimate photosynthetic parameters as accurately or better than PLSR, since LASSO is more robust when comparing different environments or plant species (Tibshirani 1996). Therefore, to bypass PLSR performance problems, we propose to explore other powerful machine learning algorithms with appropriate feature extraction capacities, which include LASSO (Vergara-Diaz et al. 2020), Bayesian Ridge (BR; Neal 1996), and Automatic Relevance Determination Regression (ARDR; Tipping 2001).

For these multivariate models, utilized data must represent enough phenotypic variability to support proper model functioning. To achieve sufficient phenotypic variability, several researchers have applied a range of growth conditions, including different levels of abiotic stresses, such as drought (Silva-Perez et al. 2017), elevated tropospheric ozone (Ainsworth et al. 2014; Yendrek et al. 2017), or high temperature (Serbin et al. 2012). Another means for increasing phenotypic variation is by including several related species in the same model. For example, Doughty et al. (2011) used 149 tropical tree species to create a PLSR model to estimate mid-day photosynthesis using canopy hyperspectral imaging; and Serbin et al. (2012) combined hyperspectral data of two tree species to estimate *V*_*c*,max_. However, to the best of our knowledge, no published study has combined multiple leguminous row crops species. In our research, we focused on soybean (*Glycine max*) and peanut (*Arachys hypogea*), which are leguminous crops often grown under high abiotic stress levels (drought and elevated temperature) in the southeastern United States. These legume crops are also important in rotation with corn and cotton.

The aims of this study were (i) to estimate photosynthetic capacity parameters, such as mid-day photosynthesis, leaf chlorophyll content (LCC), *V*_*c*,max_, and *J*_max_ of two legume crops (soybean and peanut) using full-range leaf level reflectance spectra (VIS–NIR–SWIR, 400–2500 nm) with PLSR, BR, ARDR and LASSO models and (ii) to simulate photosynthetic parameter model performance using four common types of sensors with more limited wavelength ranges: VIS–NIR (350–1000 nm), NIR–SWIR (1000–2500 nm), SWIR (1400–2500 nm), and an advanced multispectral sensor imitating the ESA Copernicus Sentinel 2 satellite with 12 spectral bands.

## Materials and methods

### Trial setup and design

Experiments were conducted in field trials and controlled conditions located at Auburn University (Alabama, USA). The study was carried out with two leguminous crops (soybean and peanut) that were exposed to different growth conditions. The first experiment involved two soybean (*Glycine max.* L) cultivars grown under ambient and elevated [CO_2_] at an Open Top Chamber Facility. The second experiment involved four soybean cultivars grown under high night temperature in growth chambers. The third experiment was performed with 6 peanut (*Arachis hypogea* L.) cultivars grown under well-watered and water-stress conditions in a greenhouse.

### Experiment 1: soybean cultivar response to elevated [CO_2_]

Two soybean cultivars representing high (PI398223) and low (PI567201A) water use efficiencies (WUE) were chosen for the study based on previous screening by Dhanapal et al. (2015). The two cultivars were planted on 16 May 2019 in 20 L pots filled with commercial growth media (Pro-Mix, Premier Tech, Quebec, Canada) at the Open Top Chamber Facility located at the USDA-ARS National Soil Dynamics Laboratory, Auburn, AL, USA. Open top chambers (OTC) (Rogers et al. 1983), encompassing 7.3 m^2^ of ground surface area, were used to deliver target [CO_2_] of ~ 410 ppm (ambient) or ambient plus 200 ppm (elevated) [CO_2_] during light hours using a delivery and monitoring system described elsewhere (Mitchell et al. 1995). There were four replicate chambers of each CO_2_ level for a total of eight experimental plots. Each OTC held two pots of each cultivar to have two sub-replicates for each plot. The experiment was conducted as a split plot design with CO_2_ level being the main plot factor and cultivar being the split plot factor. Mid-day photosynthesis and A–Ci curves were performed when plants were at the beginning of pod development (R3, Fehr et al. 1971, 15 July) and at the beginning of seed filling (R5, 26 July) according to growth stages defined by Fehr et al. (1971). Relative chlorophyll content and leaf hyperspectral reflectance measurements were performed concurrently with photosynthetic parameter measurements. More detailed information on experimental design was previously reported by Soba et al. (2020).

### Experiment 2: soybean cultivar response to high night temperatures

Four soybean cultivars (PI360846, DS25-1, PI458098, and Agx9) were planted in 3.8 L pots containing a peat-moss: perlite potting mixture (2:1) on 1 May 2019. Plants were grown at the Auburn University Plant Science Research Center greenhouse complex. Temperatures were maintained at 28/20 °C (day/night) until plants reached the first flowering stage (R1). To impose night temperature treatments, plants were then moved to two Conviron CMP 6010 growth chambers (Conviron, Manitoba, Winnipeg, Canada) maintained on a 12 h photoperiod (1200 µmol m^−2^ s^−1^ PAR) with 50/70% RH (day/night). Control plants were grown at 30/20 °C (day/night) and high night temperature plants were grown at 30/30 °C (day/night). Three replicates per cultivar and chamber were used and the whole experiment was repeated twice. Fourteen days after temperature treatments were imposed, mid-day photosynthesis, A–Ci curves, LCC, and leaf hyperspectral reflectance were performed as explained below.

### Experiment 3: peanut cultivar response to drought

Six peanut cultivars (AUG16-28, AU17, 18H19-3738, G06-G, AU8-19, and AU18-21) were planted at the Auburn University Plant Science Research Center greenhouse complex on 21 April 2019. Plants were grown in 20 L pots containing a mixture of sand and sandy-loam field soil (1:1, *w*/*w*) collected from EV-Smith Research Center, Shorter, AL, USA. Plants were maintained under well-watered conditions (80% relative soil water content, RSWC) until 60 days old; at this time, the drought experiment was initiated. Weighing pots every 2–3 days initially and every day towards the end of the experiment allowed RWSC to be gravimetrically maintained. Well-watered plants were maintained at 80% RSWC while drought plants were maintained at a 30% RSWC. Four replicates per cultivar and stress treatment were used in this experiment. At 20 and 40 days after drought initiation (i.e., 80- and 100-day-old plants), mid-day photosynthesis, A–Ci curves, LCC, and leaf hyperspectral reflectance measurements were performed as explained below.

### Physiological parameter assessments

In this study, mid-day photosynthesis, A–Ci curves, and SPAD measurements were taken from 3 different experiments and coupled with full-range (350–2500 nm), high-resolution (3–8 nm) spectral reflectance measurements taken with a Field Spec Hi-Res four field spectrometer (Analytical Spectral Devices, Boulder, CO, USA) to predict physiological parameters that characterize photosynthetic traits.

### Mid-day photosynthesis measurements

Depending on experiment size, mid-day photosynthesis measurements were taken one day before A–Ci curves using two or three LI-6400 (Li-Cor Biosciences, Lincoln, NE, USA) systems. Measurements were performed on fully expanded young leaves corresponding with the third/forth leaf from the top in soybean, and second/third leaf from the top of the main stem in peanut. Prior to measurements, systems were set to match environmental growth conditions (light intensity and temperature) and maintained at a relative humidity of 60–70%. While photosynthesis measurements were in progress, relative chlorophyll content and spectral reflectance measurements were also performed on the same leaves using a SPAD meter (Minolta SPAD-502, Spectrum Technologies Inc., Plainfield, IL, USA) and the Field Spec Hi-Res 4 field spectrometer, respectively.

### A–Ci curves

To calculate maximum rate of rubisco-catalyzed carboxylation (*V*_*c*,max_) and maximum electron transport rate supporting RuBP regeneration (*J*_max_), A–Ci curves were performed at different developmental stages in each experiment. In general, the A–Ci curves were the same for peanut and soybean except for different light saturation points: 1750 μmol m^−2^ s^−1^ PAR for soybean (Ainsworth et al. 2004) and 2000 μmol m^−2^ s^−1^ PAR for peanuts (Ferreyra et al. 2000). Photosynthesis was initially induced at the growth [CO_2_] (410 ppm for ambient and 610 ppm for elevated CO_2_ treatments), and then [CO_2_] was reduced stepwise to the lowest concentration of 50 ppm. Afterwards, [CO_2_] was increased stepwise to the highest CO_2_ concentration of 1500 ppm. A total of 11 measurements per curve were recorded (Sanz-Sáez et al. 2017). During measurements, block temperature was set at 28 °C (i.e., mean mid-day temperature at Auburn, AL). The equations and spreadsheet developed by Sharkey et al. (2007) were used to calculate *V*_*c*,max_ and J_max_ normalized at 25 °C as it has been demonstrated by (Khan et al. 2021) that different temperatures and the effect on reflectance does not affect prediction of these normalized parameters. While A–Ci curves were taken, concurrent spectral reflectance measurements were performed on the same leaves.

### Relative chlorophyll content

Relative chlorophyll content was taken on the same mid-day photosynthesis leaves using a SPAD-502 chlorophyll meter (Konica Minolta, Tokyo, Japan). Five subsample measurements per leaf were collected and averaged.

### Leaf spectral reflectance measurements

Leaf spectral reflectance was measured with a FieldSpec Hi-Res 4 concurrently on the same leaves used for photosynthetic measurements. This device has three sensors with a full spectro-radiometer range of 350–2500 nm, with a resolution of 3 nm in visible (VIS; 350–700) and near-infrared (NIR; 700–1000 nm) and 8 nm in shortwave-infrared (SWIR; 1000–2500 nm). Measurements were taken via a leaf clip coupled to a fiber-optic cable. The FieldSpec has a radiometrically calibrated internal light source, which was standardized for relative reflectance using white reference measurements every 15 min. For each leaf, 6 reflectance measurements were recorded on different regions of a single leaf per pot. We used the FieldSpectra package in R to average the six samples and align the VIS, NIR, SWIR sensors with a spectral splice correction (Serbin et al. 2014; Yendrek et al. 2017).

To accomplish the second research aim, we simulated if a more limited spectral range (corresponding to other remote sensing devices) would be able to estimate photosynthetic parameters with the same accuracy as the full-range spectra achieved with the Field Spec HiRes4. Simple spectral resampling of four different sensors was performed to simulate commercial spectrophotometer sensors, such as the UniSpec-DC VIS/NIR (310–1100 nm; PP Systems, Amesbury, MA, USA), the USB 2000 VIS/NIR (340–1014 nm; Ocean Optics, Dunedin, FL, USA), and the Liga SWIR spectrophotometer (850–1888 nm; STEAG Micro Parts, Dortmund, Germany). We also included a resampling simulation for the bands and bandwidths of the ESA Copernicus Sentinel-2 satellite, with 12 spectral bands (443, 494, 560, 665, 704, 740, 781, 834, 944, 1375, 1612, and 2194 nm) representing VIS, NIR, and SWIR (see more in Drusch et al. 2012; Segarra et al. 2020).

### Statistical analysis of measured and estimate values

Statistical analyses were conducted using R Studio (RStudio Team 2020) and Python 3.7 (Python Software Foundation, https://www.python.org) via a Jupiter notebook (Wofford et al. 2019). Effects of abiotic stress treatments and differences between cultivars on studied variables were assessed using analysis of variance (ANOVA) in R Studio. We also analyzed correlations between photosynthetic parameters against each spectrum band by Pearson’s correlation using R Studio.

With respect to the different advance regression models, we used the SciPy module (Jones et al. 2001; Varoquaux et al. 2015) in Python 3.7 and the Scikit-Learn library for the estimation of different parameters to estimate determination (*R*^2^) and the root means squared error (RMSE). For cross-validation, we used the “train test split method” where, we split our data into training (60% of the data used to build the model) and testing (40% of the data used to test the model). This method quantifies the prediction error, the RMSE, which measures the average prediction error made by the model in predicting the outcome for an observation. That is, the average difference between the observed known outcome values and the values predicted by the model. Associations between photosynthetic parameters (response variables) and the leaf reflectance spectrum (explanatory) variables were analyzed using four advances models: (i) Partial Least Squares Regression (PLSR) is based on the dimension reduction method (Wold et al. 2001). For this model, we used between 5 and 11 components, choosing the number of components that gave the highest *R*^2^ and the lower RSME; (ii) Least Absolute Shrinkage and Selection Operator (LASSO) is a shrinkage method (Tibshirani 1996); (iii) Bayesian ridge (BR) and (iv) Automatic relevance determination regression (ARDR) are both high-dimensional methods (Neal 1996; Tipping 2001). Figures were prepared using the matplotlib (Hunt 2019) and Seaborn Python (Waskom et al. 2017) modules in Python 3.7.

## Results

### Effect of abiotic stress and cultivar on photosynthetic parameters

Analyzing the effect of abiotic stress and cultivars can yield valuable insights into phenotypic range of variation within each experiment. In Experiment 1, the two soybean cultivars showed significant effects of [CO_2_] on mid-day photosynthesis and LCC, but not on *V*_*c*,max_ and *J*_max_ (Table 1 and Fig. S2). We observed treatment effects for mid-day photosynthesis and LCC (Table 1a). In summary, phenotypic variation was noticeable with a range of 17.01–36.22 µmol m^−2^ s^−1^ for mid-day photosynthesis, 34.55–51.35 for LCC, 182.9–348.4 µmol m^−2^ s^−1^ for *V*_*c*,max_, 174.7–263.7 µmol m^−2^ s^−1^ for *J*_max_, and 29.4–30.37 °C for leaf temperature. In Experiment 2, four soybean cultivars were grown under high night temperature (30/30 °C day/night) for comparison to controls (30/20 °C day/night). Cultivar effects with treatment showed a significant effect on mid-day photosynthesis, LCC, and *J*_max_ (Table 1b). Overall, phenotypic variation was noticeable with a range of 11.52–32.68 µmol m^−2^ s^−1^ for mid-day photosynthesis, 34.01–53.95 for LCC, 48.01–135.2 µmol m^−2^ s^−1^ for *V*_*c*,max_, 61.01–165.1 µmol m^−2^ s^−1^for *J*_max_, and 29.9–30.33 °C for leaf temperature. In Experiment 3, the effect of drought was significant for all measured peanut parameters except for *V*_*c*,max_ and *J*_max_ (Table 2). Cultivars only showed significant effects for LCC and *J*_max_. The interaction effects of drought and cultivars was only slightly significant for *V*_*c*,max_ (*P* = 0.094). Phenotypic variation was perceptible since mid-day photosynthesis ranged from 5.051 to 26.41 µmol m^−2^ s^−1^, LCC varied from 42.30 to 52.45, *V*_*c*,max_ varied from 64.38 to 171.3 µmol m^−2^ s^−1^, *J*_max_ ranged from 79.3 to 206.1 µmol m^−2^ s^−1^, and 28.6 to 30.5 °C for leaf temperature. When phenotypic variation of all three experiments was considered together, the range for mid-day photosynthesis was 5.051–36.22 µmol m^−2^ s^−1^, 34.55–53.95 for LCC, 48.01–348.4 µmol m^−2^ s^−1^ for *V*_*c*,max_, 61.01–263.7 µmol m^−2^ s^−1^ for *J*_max_, and 26.33–31.55 °C for leaf temperature (Fig. S2, shows the Box plot for each experiment).

**Table 1:** Mean values of mid-day photosynthesis (µmol m^−2^ s^−1^), leaf chlorophyll content (LCC, arbitrary units), maximum rate of rubisco-catalyzed carboxylation (V_c,max_, µmol m^−2^ s^−1^), maximum electron transport rate supporting RuBP regeneration (J_max_, µmol m^−2^ s^−1^), and leaf temperature (°C) per each treatment. (**a**) Experiment 1: two varieties of soybean grown at 410 ppm and 610 ppm of [CO_2_]; *n* = 32. (**b**) Experiment 2: four soybean varieties grown at low (20 °C) and high (30 °C) night temperature; *n* = 48

(A)						
Genotype	Treatment	Photosynthesis (µmol m^−2^ s^−1^)	LCC (arbitrary unit)	Vcmax (µmol m^−2^ s^−1^)	Jmax (µmol m^−2^ s^−1^)	Leaf Temperature (°C)
Pi398223	410	23.3 ± 3.6 b	45.5 ± 3.6 a	249.5 ± 44.5 ab	212.2 ± 23.3 a	30.31 ± 0.388 a
Pi567201	410	25.1 ± 3.9 b	41.6 ± 3.3 b	269.8 ± 50.5 a	227.8 ± 31.9 a	30.06 ± 0.708 a
Pi398223	610	30.4 ± 2.1 a	46.3 ± 3.5 a	227.2 ± 22.7 b	211.4 ± 19.7 a	30.37 ± 0.706 a
Pi567201	610	31.2 ± 4.8 a	46.7 ± 2.2 a	257.4 ± 40.9 ab	219.2 ± 16.2 a	29.42 ± 1.377 a
ANOVA	**[CO**_**2**_**]**	0.001***	0.15*	*0.242*	*0.569*	*0.354*
ANOVA	**Varieties**	*0.344*	*0.13*	0.092·	*0.173*	0.06·
ANOVA	**[CO**_**2**_]*Varieties	*0.678*	0.077·	*0.733*	*0.646*	*0.265*

(B)						
Genotype	Treatment	Photosynthesis (µmol m^−2^ s^−1^)	LCC (arbitrary unit)	Vcmax (µmol m^−2^ s^−1^)	Jmax (µmol m^−2^ s^−1^)	Leaf Temperature (°C)
PI360846	Low T	17.7 ± 5.2 b	45.1 ± 0.6 ab	72.7 ± 28.6 a	102.6 ± 33.7 bc	30.06 ± 0.059 ab
PI458098	Low T	21.4 ± 4.8 ab	49.8 ± 3.7 a	91.7 ± 37.2 a	128.6 ± 43.1 ab	29.98 ± 0.418 ab
DS25-1	Low T	22.2 ± 1.3 ab	38.4 ± 3.9 c	82.7 ± 9.5 a	111.0 ± 10.2 abc	29.86 ± 0.450 b
AG48 × 9	Low T	27.2 ± 3.9 a	45.9 ± 1.5 a	107.6 ± 13.1 a	149.0 ± 16.6 a	29.98 ± 0.216 ab
PI360846	High T	16.9 ± 5.3 b	47.3 ± 4.7 a	100.6 ± 29.1 a	136.3 ± 34.3 ab	30.33 ± 0.170 a
PI458098	High T	16.9 ± 0.5 b	50.1 ± 4.1 a	84.7 ± 29.7 a	108.0 ± 14.7 abc	29.91 ± 0.188 ab
DS25-1	High T	15.6 ± 3.6 b	39.7 ± 3.2 bc	68.7 ± 7.4 a	78.67 ± 16.3 c	30.08 ± 0.202 ab
AG48 × 9	High T	27.5 ± 5.1 a	45.7 ± 3.2 a	109.3 ± 26.0 a	146.6 ± 25.2 ab	30.01 ± 0.146 ab
ANOVA	**Temperature**	*44.81*	*0.522*	*0.833*	*0.624*	*0.312*
ANOVA	**Varieties**	0.010**	0.002**	*0.303*	0.042*	*0.561*
ANOVA	Temp*Varieties	*0.999*	*0.999*	*0.999*	*0.999*	*0.999*

Levels of significance: *x*, *P* < 0.1; *P < 0.05; **P < 0.01; ***P < 0.001

**Table 2:** Mean values of midday photosynthesis (µmol m^−2^ s^−1^), leaf chlorophyll content (LCC, arbitrary units), maximum rate of rubisco-catalyzed carboxylation (*V*_*c*,max_, µmol m^−2^ s^−1^), maximum electron transport rate supporting RuBP regeneration (*J*_max_, µmol m^−2^ s^−1^), and leaf temperature (°C) in six varieties of peanut grown under well-watered (WW, 80% SWC) and water-stress (WS, 30% SWC) conditions

Genotype	Treartment	Photosynthesis (µmol m^−2^ s^−1^)	LCC (arbitrary unit)	*V*_*c*,max_ (µmol m^−2^ s^−1^)	*J*_max_ (µmol m^−2^ s^−1^)	Leaf Temperature (°C)
18H19-3738	WW	22.2 ± 2.1 a	50.7 ± 1.4 bcd	126.2 ± 17.1 a	169.2 ± 14.9 ab	28.93 ± 0.906 ab
AU17	WW	21.1 ± 4.8 a	49.6 ± 1.9 cde	135.9 ± 19.9 a	179.9 ± 29.8 a	28.67 ± 0.727 b
AU18-21	WW	21.1 ± 1.9 ab	45.7 ± 0.8 e	129.6 ± 15.1 a	159.5 ± 31.4 abc	29.96 ± 0.662 ab
AU16-28	WW	20.2 ± 4.8 ab	46.6 ± 3.6 de	136.1 ± 20.4 a	183.4 ± 18.9 a	29.01 ± 0.974 ab
AU18-19	WW	17.9 ± 2.5 abc	46.9 ± 2.7 de	108.9 ± 19.2 abc	150.2 ± 22.6 abc	29.58 ± 1.377 ab
G-06-G	WW	17.9 ± 5.0 abc	45.6 ± 2.2 e	123.1 ± 35.2 ab	139.8 ± 45.1 bcd	29.37 ± 1.489 ab
18H19-3738	WS	15.4 ± 3.5 bcd	56.6 ± 1.2 a	125.7 ± 40.4 ab	153.94 ± 31.5 abc	30.40 ± 0.640 ab
AU17	WS	14.1 ± 2.1 cd	53.1 ± 4.6 abc	131.1 ± 22.6 a	152.3 ± 5.8 abc	29.24 ± 0.688 ab
AU18-21	WS	10.7 ± 5.4 d	52.1 ± 4.1 abc	85.4 ± 32.8 c	109.1 ± 22.8 d	29.24 ± 0.688 ab
AU16-28	WS	12.5 ± 2.8 d	54.9 ± 2.7 ab	113.7 ± 14.6 abc	121.3 ± 26.7 cd	30.56 ± 0.707 a
AU18-19	WS	11.9 ± 3.2 d	54.8 ± 4.1 ab	135.4 ± 15.9 a	133.6 ± 37.3 bcd	30.58 ± 0.224 a
G-06-G	WS	10.3 ± 1.8 d	49.3 ± 2.8 cde	89.1 ± 25.8 bc	126.7 ± 15.9 cd	29.47 ± 1.351 ab
ANOVA	**Drought**	0.001***	0.001***	0.46	0.275	0.02*
ANOVA	**Varieties**	0.154	0.001***	0.196	0.092·	0.837
ANOVA	Drought*Varieties	0.884	0.353	0.094·	0.352	0.461

Levels of significance: *x*, *P* < 0.1; *P < 0.05; **P < 0.01; ***P < 0.001; *n* = 48

### Relationships between spectral signatures and photosynthetic parameters

**Fig. 1:**
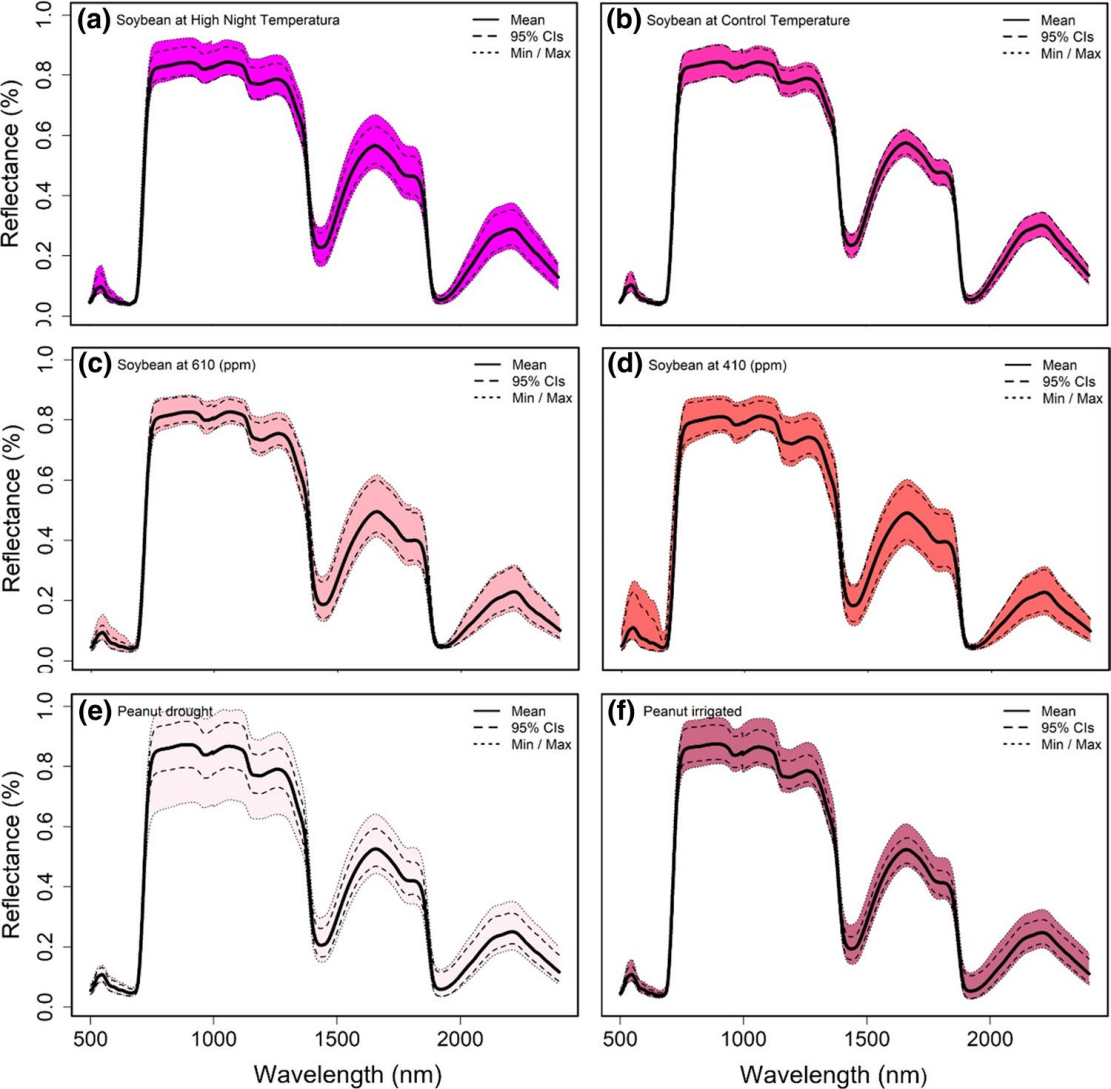
**a** Mean, ± standard deviation (*n* = 24), and minimum and maximum leaf reflectance for soybean at high night temperature grown in growth chambers. **b** Mean, ± standard deviation (*n* = 24), and minimum and maximum leaf reflectance for soybean at control temperature grown in growth chambers. **c** Mean, ± standard deviation (*n* = 18), and minimum and maximum leaf reflectance for soybean at 610 ppm grown at an Open Top Chamber Facility. **d** Mean, ± standard deviation (*n* = 18), and minimum and maximum leaf reflectance for soybean at 410 ppm grown at an Open Top Chamber Facility. **e** Mean, ± standard deviation (*n* = 24), and minimum and maximum leaf reflectance for peanut drought grown under greenhouse conditions. **f** Mean, ± standard deviation (*n* = 24), and minimum and maximum leaf reflectance for peanut irrigated grown under greenhouse conditions

Figure 1 presents the sensitivity of leaf reflectance spectrum for different species and abiotic stresses. Under high night temperature, soybean reflectance spectrum shows higher variability than the control with a larger peak at ~ 550 nm and wider reflectance band between ~ 750–1400, 1550–1800 and 2000–2300 nm (Fig. 1a, b). Elevated CO_2_ in soybean tended to reduce variability of the reflectance spectrum between ~ 500–600 and 750–1400 while maintaining the variability in the reflectance spectrum between 1550–1800 and 2000–2300 nm (Fig. 1c, d). In peanut, drought increased variability at all wavelengths with the exception of the 500–600 nm range (Fig. 1e, f). When comparing reflectance of the two legume species, we noted that peanut added a lot of spectral variation in the range from 750 to 2300 nm, probably due to the drought treatment; meanwhile soybean added more variability in the 500–600 nm range (Fig. S1).

**Fig. 2:**
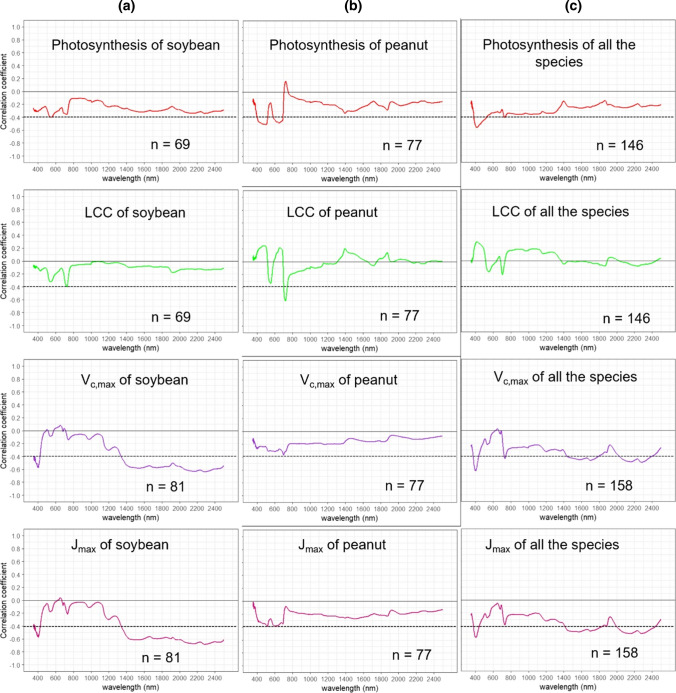
Pearson’s correlation coefficients (r) between photosynthetic parameters and each wavelength from the leaf reflectance spectrum for each species and both species combined. **a** Soybean varieties under two treatments, one at high [CO_2_] and the other at high temperature. **b** Peanut varieties at water stress. **c** Soybean and peanut data pooled together. Each graphic presents in the x-axis the wavelength spectrum between 350 and 2500 nm and in the y-axis the Pearson’s correlation coefficient from − 1 to 1. The discontinuous line in each graphic means the significance level *P* < 0.05 below the x-axis

Pearson’s correlations were performed to highlight which zones of spectral signatures presented negative or positive correlations with each measured parameter. Pearson’s correlations between the parameter and each wavelength were presented separately for soybean (Fig. 2a), peanut (Fig. 2b), and both species combined (Fig. 2c). Regarding soybean *V*_*c*,max_ and *J*_max_ values, correlation against each band showed significant (*P* < 0.05) negative values (Pearson coefficient around − 0.6) in the VIS (400 nm) and in almost all SWIR (1400–2500 nm) bands (Fig. 2a). On the other hand, mid-day photosynthesis and LCC presented lower and no significant correlation coefficients against each band from the reflectance spectrum. In the case of peanut (Fig. 2b), photosynthesis values against each wavelength band showed significant correlation (*r* = − 0.6, *P* < 0.05) in VIS–NIR (400–1000 nm) bands. LCC and each wavelength showed strong correlation (*r* = − 0.7, *P* < 0.05) in the NIR (700 nm). For *V*_*c*,max_ and *J*_max_, the correlation against each wavelength was very low or non-significant (Fig. 2b). With increased variability from combining all experiments, we could observe that mid-day photosynthesis against each wavelength showed a significant correlation (*r* = − 0.5, *P* < 0.05) in the VIS (400 nm). Regarding the coefficient of correlation between *V*_*c*,max_ and *J*_max_, significance (*r* = 0.6, *P* < 0.05) in the VIS (400 nm) and most of the SWIR (1400–2500 nm) bands indicated an improvement relative to species analyzed separately. For this reason, we ran all advance models using combined phenotypic and spectral data from each species and environmental condition.

### Estimating photosynthetic parameters using field spectroscopy and advance regression models

**Fig. 3:**
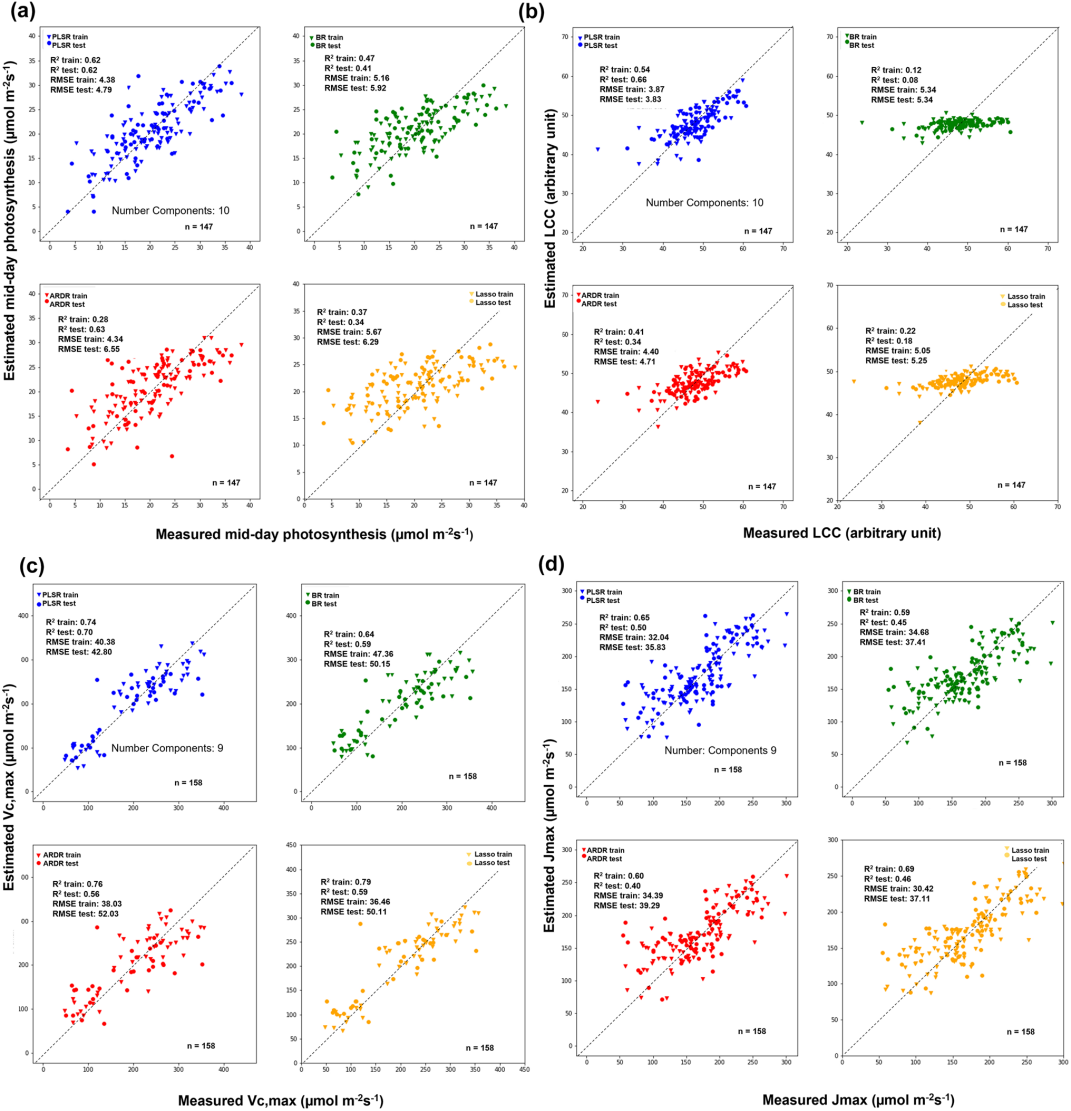
Measured against estimated values correlation for different physiological parameters estimated with PLSR (blue), BR (green), ARDR (red), and LASSO (yellow) predictive models. The estimated physiological parameters are: mid-day photosynthesis (**a**), leaf chlorophyll content (**b**), maximum rate of Rubisco catalyzed carboxylation (Vc,max, **c**) and maximum electron transport rate supporting RuBP regeneration (Jmax, **d**) for soybean and peanut cultivars all pooled together. All the models were built using train and test data splitting them into 60 and 40%, respectively. In each graph, the *R*^2^, the RMSE of the train and test of the model are shown along with the size of the train and test population and number of model components (comp) used in each PLSR model. The gray dashed line shows the 1:1 line

To test how accurately a given model estimated different photosynthetic parameters, we presented the coefficient of determination (*R*^2^) and RMSE for each model and mean parameter, i.e., interpreted as the proportion of information in data that is explained by each model (Fig. 3). Since estimation of the *V*_*c*,max_ and *J*_max_ parameters did not work well in the peanut experiment but worked well for the soybean (Table S1), and since the LCC estimation does not work with soybean, we decided to combine these three experiments and focus on the combination of the two crop species in this manuscript (Fig. 3). Mid-day photosynthesis showed a higher *R*^2^ (0.62) and low RMSE (4.79) using the PLSR model using 10 components, followed by BR (*R*^2^ = 0.41 and RMSE = 5.92) with the worst model being the ARDR (*R*^2^ = 0.28 and RMSE = 6.55) (Fig. 3a). LCC was better assessed by PLSR (*R*^2^ = 0.56 and RMSE 3.83) using 10 components, followed by ARDR (*R*^2^ = 0.34 and RMSE = 4.71) with the BR model showing the worst performance (*R*^2^ = 0.08 and RMSE = 5.55; Fig. 2b). The best *V*_*c*,max_ model was obtained by PLSR (*R*^2^ = 0.70 and RMSE = 42.80) using nine components followed by the other three models with similar values (*R*^2^ = 0.56–0.59; RMSE = 50.11–52.03). Regarding *J*_max_, the best model was PLSR (*R*^2^ = 0.50 and RMSE = 35.83) using nine components closely followed by Lasso (*R*^2^ = 0.46 and RMSE = 37.1) and BR (*R*^2^ = 0.45 and RMSE = 37.41), with ARDR (*R*^2^ = 0.40 and RMSE = 39.29) being the worst model.

**Fig. 4:**
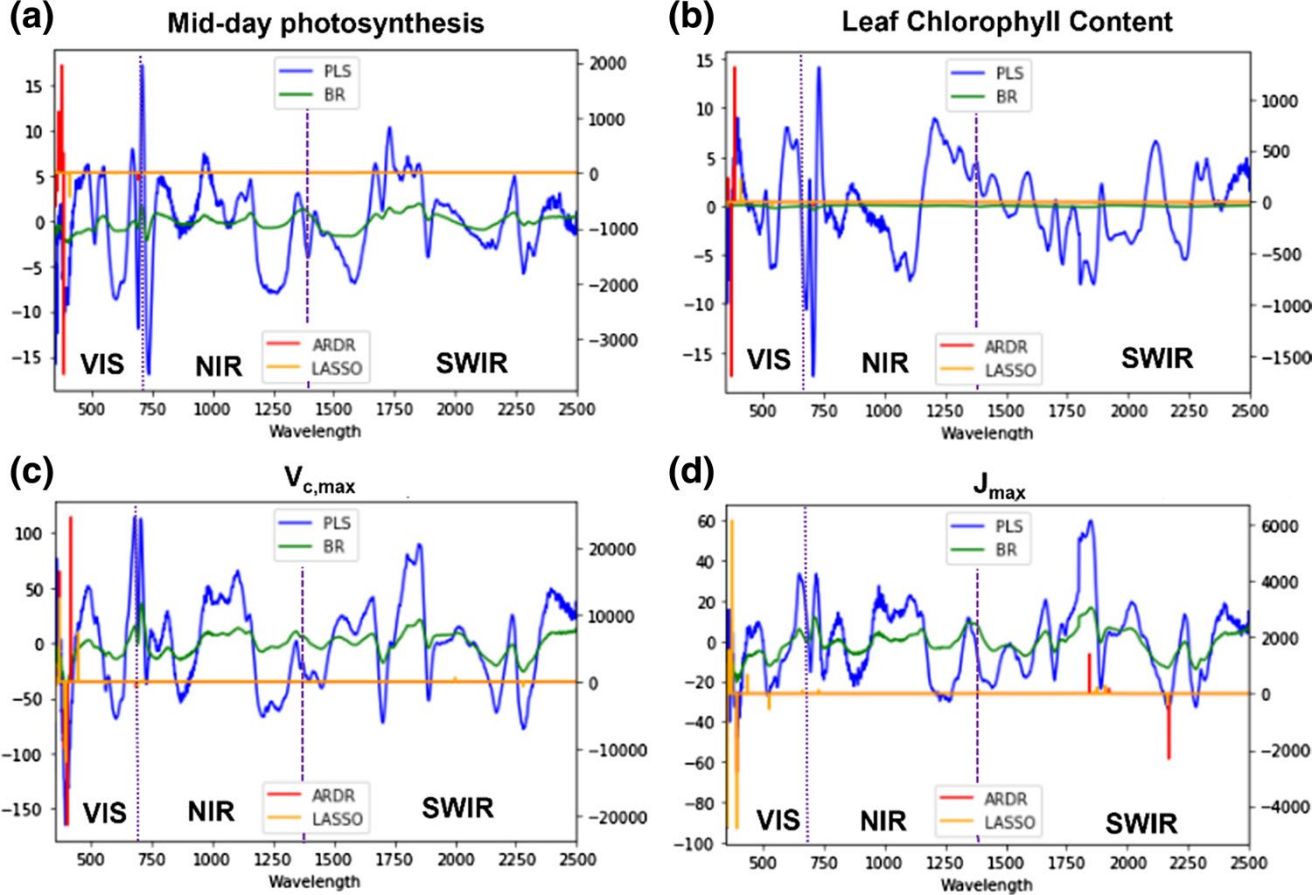
Spectral-specific coefficients for each prediction model (PLSR, BR, ARDR and LASSO) used to predict the following photosynthetic parameters of the two species pooled together. **a** Mid-day photosynthesis. **b** Leaf chlorophyll content (LCC). **c** Maximum rate of Rubisco carboxylation (*V*_*c*,max_). **d** Maximum electron transport rate supporting RuBP regeneration (*J*_max_,). Continuous vertical lines delineate different regions of the spectrum: VIS = 450–700 nm, NIR = 700–1400, and SWIR = 1400–2500 nm

For each of the four models, we calculated the coefficient of weight for each band and model (Fig. 4). These coefficients showed waveband contributions along the VIS–NIR–SWIR spectrum for photosynthetic parameter estimations using leaf reflectance spectrum of pooled species, cultivars, and growing conditions. The coefficient of weight for estimating mid-day photosynthesis using PLSR showed maximum values around 400, 750, and 1750 nm, while ARDR and LASSO showed high coefficient weights at 400 nm. On the other hand, BR did not show any remarkable coefficient weights for mid-day photosynthesis (Fig. 4a). With respect to LCC, PLSR showed maximum coefficients at 400, 750, and 1750 nm, while ARDR showed a peak around 400 nm (Fig. 4b). LASSO and BR showed very low coefficients at all wavelengths (Fig. 4b). In Fig. 4c, we can observe the different coefficients of each band for *V*_*c*,max_, where the maximum peaks were at 400, 700 and around 2000 nm for PLSR, BR and LASSO, while for ARDR it was only at 400 and 750 nm. For estimates of *J*_max_, the highest coefficient weights for PLSR were located in SWIR (2200–2300), followed by NIR (900–1100). For the LASSO model, the strongest areas were at 400, 750, and 1750 nm (Fig. 4d), while the highest coefficients were found in the SWIR (1400–2500 nm) for BR and ARDR.

### Scaling up estimations of photosynthetic parameters for potential hyperspectral aerial or satellite applications

To assess their ability to estimate photosynthetic parameters compared to full spectra captured by the Field Spec Hi-Res4 (VIS–NIR–SWIR, 350–2500 nm), we simulated other sensors with limited wavelength ranges, specifically VIS–NIR (350–1000 nm), NIR–SWIR (1000–2500 nm), SWIR (1400–2500 nm), and the 12 wavelength bands of Sentinel-2 satellites (Table S2). To test this, we used reflectance data acquired by the Field Spec Hi-Res4 and separated the reflectance data according to the wavelength range of each before mentioned sensor. We then performed photosynthetic estimations using the same 4 models (PLSR, BR, ARDR, and LASSO).

**Fig. 5:**
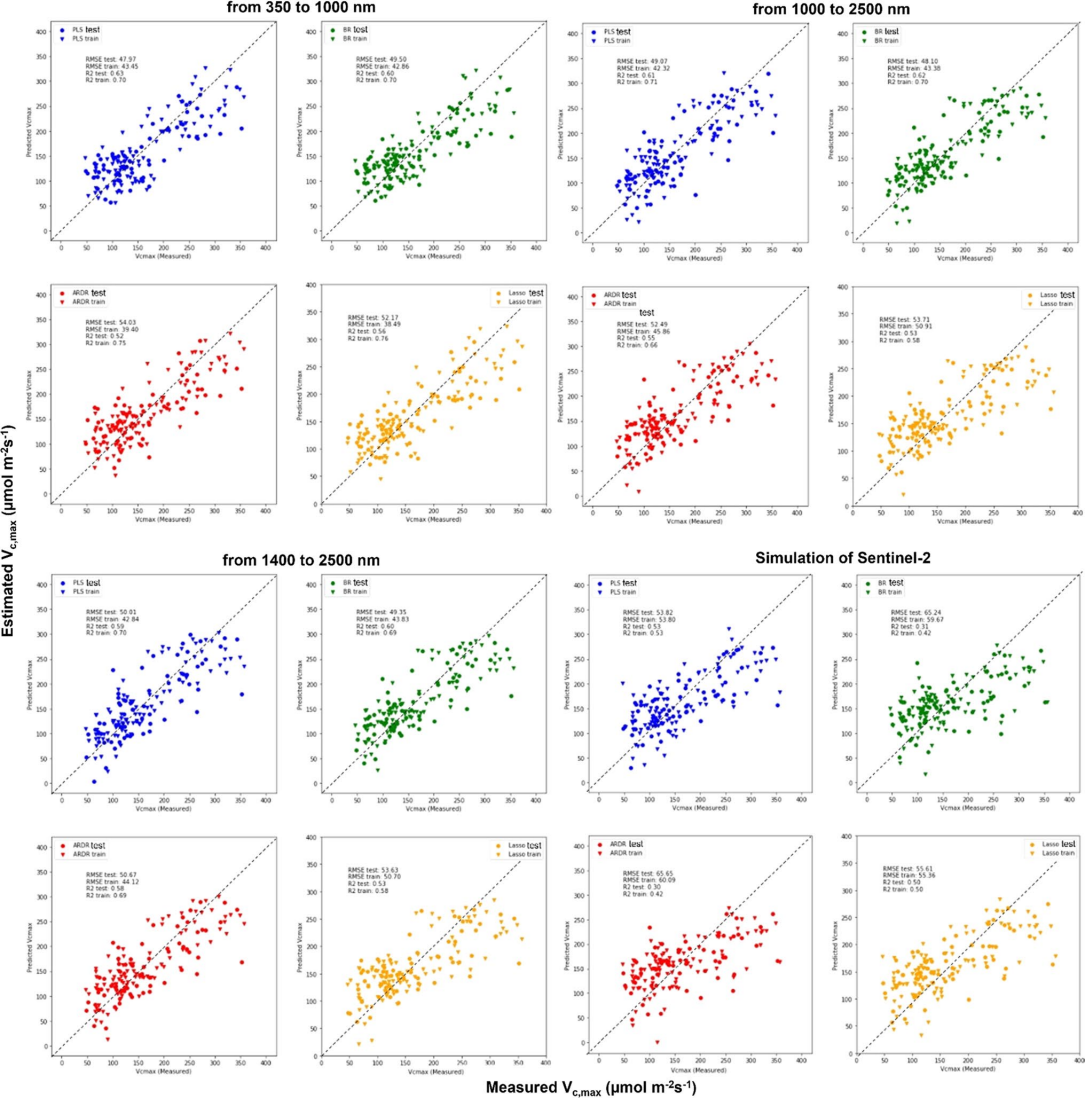
Measured (*X*-axis) against estimated (*Y*-axis) correlation of maximum rate of rubisco-catalyzed carboxylation (Vc,max) estimated with PLSR (blue), BR (green), ARDR (red), and LASSO (yellow) predictive models. These models were based on leaf reflectance spectra at different ranges [VIS–NIR (350–1000 nm), NIR–SWIR (1000–2500 nm), SWIR (1400–2500 nm), and Sentinel-2 bands] for soybean and peanut cultivars all pooled together. All the models were built using the training and test split method (60 and 40%, respectively). Each graph shows the train and test *R*^2^ and the RMSE values of for each model. For PLSR models, we used 10 components. Size of population is *n* = 158. The gray dashed line shows the 1:1 line

**Fig. 6:**
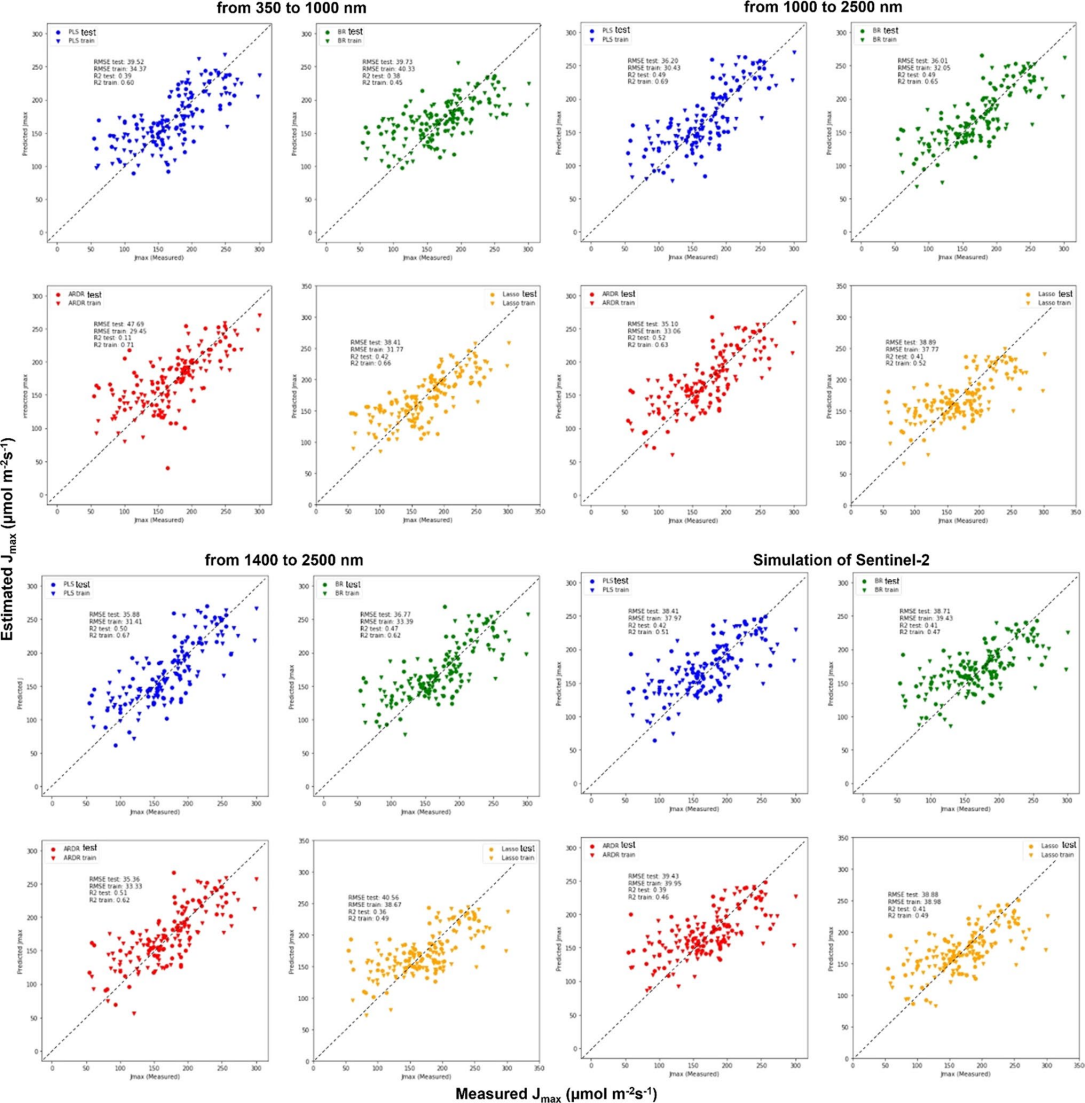
Measured (axis * X*) against estimated (axis * Y*) correlation of maximum electron transport rate supporting RuBP regeneration (*J*_max_) estimated with PLSR (blue), BR (green), ARDR (red), and LASSO (yellow) predictive models. These models were based on leaf reflectance spectra at different ranges [VIS–NIR (350–1000 nm), NIR–SWIR (1000–2500 nm), SWIR (1400–2500 nm), and Sentinel-2 bands] for soybean and peanut cultivars all pooled together. All the models were built using the training and test split method (60 and 40%, respectively). Each graph shows the train and test * R*^2^ and the RMSE values of for each model. For PLSR models, we used 10 components. Size of population is *n* = 158. The gray dashed line shows the 1:1 line

Table 3, Figs. 5, and 6 show estimations of photosynthetic parameters using pooled data from both species. For mid-day photosynthesis and LCC, simulations with different sensors with just the VIS–NIR (350–1000 nm), NIR–SWIR (1000–2500 nm), and SWIR (1400–2500 nm) spectrum regions were best performed using PLSR compared to BR, ARDR, and LASSO models (Table 3; Figs. 5, 6). However, LCC was estimated best by BR, ARDR, and LASSO using the simulated ESA Copernicus Sentinel-2 satellite multispectral bands (Table 3; Figs. 5, 6). Concerning estimation of *V*_*c*,max_ within the VIS–NIR range (350–1000 nm) and the ESA Copernicus Sentinel-2 satellite sensors, the best performing model was PLSR using 10 components (*R*^2^ = 0.63 and 0.53, respectively). For simulations of the NIR–SWIR (1000–2500 nm), SWIR (1400–2500 nm), BR was the best model for assessing *V*_*c*,max_ (*R*^2^ = 0.62 and 0.60, respectively). For estimating *J*_max_ with the VIS–NIR (350–1000 nm) sensor, the best model was LASSO (*R*^2^ = 0.42). For the range NIR–SWIR (1000–2500 nm), SWIR (1400–2500 nm) ARDR estimated *J*_max_ similarly (*R*^2^ = 0.51). PLSR, BR, and LASSO presented the same coefficient of determination (*R*^2^ = 0.41) when using ESA Copernicus Sentinel-2 satellite simulated wavebands to assess *J*_max_.

**Table 3:** Coefficient of determination (*R*^2^) and root mean squared error (RMSE) of mid-day photosynthesis (µmol m^−2^ s^−1^), leaf chlorophyll content (arbitrary units) of all species pooled together based on leaf reflectance spectra at different ranges [VIS–NIR (350–1000 nm), NIR-SWIR (1000-–2500 nm), SWIR (1400–2500 nm), and Sentinel-2 bands] through advance regression models: Partial Least Squares Regression (PLSR), Bayesian Ridge (BR), the Automatic Relevance Determination Regression (ARDR), and Least Absolute Shrinkage and Selection Operator (LASSO)

Estimation using the species, cultivars, and growing conditions together								
*n* = 146	From 350 to 1000 nm		From 1000 to 2500 nm		From 1400 to 2500 nm		Simulation of Sentinel-2	
Model	*R*^2^	RMSE	*R*^2^	RMSE	*R*^2^	RMSE	*R*^2^	RMSE
Mid-day photosynthesis								
PLSR	0.47	5.61	0.42	6.1	0.52	5.53	–	–
BR	0.28	6.54	0.47	5.84	0.50	5.69	–	–
ARDR	0.27	6.59	0.40	6.20	0.49	5.70	–	–
LASSO	0.34	6.29	–	–	–	–	–	–
Leaf chlorophyll content								
PLSR	0.35	4.98	0.33	4.09	0.22	4.41	–	–
BR	0.26	4.98	–	–	–	–	0.23	7.04
ARDR	–	–	–	–	0.33	40.90	0.26	6.90
LASSO	0.15	5.33	–	–	–	–	0.28	6.79

“–” indicates that the estimation model did not yield results

Regarding comparison of different sensors (VIS–NIR, NIR–SWIR, and SWIR) against original FieldSpec data (VIS–NIR–SWIR), we observed that estimation of mid-day photosynthesis by the different models was similar to that of simulated sensors (Figs. 3, 5, 6 and Table 3). With ESA Copernicus Sentinel-2 satellite, estimation of mid-day photosynthesis did not work. Estimation of LCC using ESA Copernicus Sentinel-2 satellite was lower than when the whole spectrum was used. Regarding the estimation of the *V*_*c*,max_ simulating ESA Copernicus Sentinel-2 satellite, the PLSR and LASSO presented an *R*^2^ (0.50) that was a little lower than the FieldSpec (*R*^2^ = 0.70). With respect to *J*_max_ estimation, we observed that coefficients for the simulated NIR–SWIR and SWIR sensor ranges were very similar (but slightly lower) to the full-range FieldSpec (Figs. 3, 5, 6 and Table 3). The VIS–NIR and ESA Copernicus Sentinel-2 satellite simulations presented values that were lower than using the whole spectrum (Figs. 3, 5, 6 and Table 3).

## Discussion

### Estimating photosynthetic parameters using field spectroscopy and advance regression models

The main objective of this research was to assess which advanced statistical model (PLSR, BR, ARDR, and LASSO) was the most successful in estimating different photosynthetic parameters using leaf reflectance spectra (VIS–NIR–SWIR, 350–2500 nm) from two legume species. The use of advance regression models to predict different physiological parameters needs ample phenotypic variation to be accurate (Kuhn and Johnson 2013). Since statistical effects of different treatments over some variables were not significant (Table 1), we combined findings from three experiments (two different species) to increase phenotypic range for better parameter estimation with all models rather than examining each species separately (Table S1). Similar approaches have been used recently to increase phenotypic variation and obtain a better prediction model by including different species and/or cultivars (Doughty et al. 2011; Serbin et al. 2012; Choquette et al. 2019), different abiotic stresses such as drought (Silva-Perez et al. 2018), or elevated atmospheric ozone concentrations (Ainsworth et al. 2014; Yendrek et al. 2017).

In our study, when data from both legumes were combined, almost all of the advanced models were able to estimate *V*_*c*,max_ and *J*_max_ at greater than *R*^2^ > 0.50 (Fig. 3). Of the four models used to predict these two parameters, PLSR was the overall best model for *V*_*c*,max_ (*R*^2^ = 0.70 and RMSE 42.80) and *J*_max_ (*R*^2^ = 0.50 and 35.83), followed by LASSO and BR for *V*_*c*,max_ (*R*^2^ = 0.59 with RMSE 50.11; 0.59 with a RMSE 50.15, respectively), and BR and LASSO for *J*_max_ (*R*^2^ = 0.45 with a RMSE 37.11; 0.46 with a RMSE 37.41, respectively) (Fig. 3). This may be because the PLSR model does not estimate shrinkage when performing variable selection (spectral wavebands) as do BR, ARDR, and LASSO (Neal 1996; Tipping 2001; Wold et al. 2001). Others have also found that PLSR and LASSO had similar estimation capacities, showing that LASSO band block contribution was similar to the PLSR model (Fu et al. 2020). Specific reasons why PLSR was more efficient at estimating photosynthetic parameters assessed in this study are discussed in detail below.

Successful predictions of *V*_*c*,max_ (*R*^2^ = 0.89 with a RMSE 15.4) and *J*_max_ (*R*^2^ = 0.93 with a RMSE 18.67) using PLSR have been previously obtained by combining two tree species (Serbin et al. 2012); this study showed statistically significant phenotypic variation due to temperature treatments as well as species. In our study, the lower *R*^2^ associated with *V*_*c*,max_ and *J*_max_ estimates could be attributed to the lack of effect of some environmental treatments (temperature, elevated CO_2_, and drought) and cultivars over these parameters (Table 1). However, Ainsworth et al. (2014) showed a significant correlation between measured and estimated *V*_*c*,max_ (*R*^2^ = 0.88 with a RMSE 13.4) with the effect of treatments (elevated ozone) and cultivars not being significant. This demonstrated that good parameter estimation and significant treatment or cultivar effects are not mutually exclusive and that it is only necessary to have sufficient range in variation of phenotypic data. For example, Ainsworth et al. (2014) and Serbin et al. (2012) noted *V*_*c*,max_ variation (60–280 μmol m^−2^ s^−1^ and 40–170 μmol m^−2^ s^−1^, respectively) similar to the values obtained in this study when all three experiments were combined (48–348 μmol m^−2^ s^−1^ for the current experiment). Since the ranges in variation of *V*_*c*,max_ and *J*_max_ data are similar but higher to those obtained in the above-mentioned research, why are *R*^2^ values in the current study for *V*_*c*,max_ (*R*^2^ = 0.70) and *J*_max_ (*R*^2^ = 0.50) lower and RSME (42.80 and 35.83, respectively) higher than in those studies? Tibshirani (1996) has noted that PLSR models lose accuracy when estimating parameters across different environments. Research by Serbin et al. (2012) and Ainsworth et al. (2014) were each performed in one environment (greenhouse and field, respectively) for one growing season, while our study combined information from three experiments representing distinct environments (greenhouse, growth chambers, and open top chambers) with plants grown at very different environmental conditions. In an experiment with several corn breeding lines grown under ambient and elevated ozone repeated over three growing seasons, Yendrek et al. (2017) obtained *V*_*c*,max_ estimations (*R*^2^ = 0.55 with RMSE 6.61, and 0.65 with a RMSE 6.60) similar to those reported in our study but with a RMSE lower than ours. This was probably due to the effects of changing environments on PLSR performance (Serbin et al. 2012; Ainsworth et al. 2014). Regarding the lower RMSE obtained in the above-mentioned publications (Serbin et al. 2012; Ainsworth et al. 2014; Yendrek et al. 2017) in comparison with those obtained in our research, this could be due to the different cross-validation used in our approach. In our cross-validation, the test error rate can be highly variable, depending on which observations are included in the training set and which observations are included in the validation set. This may be the reason for the higher RMSE values observed in *V*_*c*,max_ and *J*_max_. Also the high RMSE values can be due to a higher phenotypic range as a result of including two crop species grown in three very different environments. This highlights the importance of performing calibration experiments under multiple environments. Other issue that can arise is the use of these models with completely new set of cultivars and experimental conditions as was tested in Yendrek et al. (2017). In such a case, it would be recommendable to test model precision by measuring spectral reflectance under new conditions and corroborating model estimates of extreme values for *V*_*c*,max_ with ground truth measurements of the photosynthetic parameter. Although this extra step will take more time, this procedure could serve to test model accuracy and help improve the model with new training data.

To solve this multiple environment/location problem, new approaches need to be developed and implemented. For example, Fu et al. (2020) increased prediction model accuracy by stacking different machine learning algorithms (i.e., *R*^2^ increases of 0.1–0.2 over single prediction models). Another alternative would be creation of a consortium of scientists interested in using hyperspectral reflectance technology to predict physiological traits. Their combined expertise would create strong standardized calibrations that could be used across multiple environments as has been done for assessing forage quality traits using NIRS technology (i.e., NIRS Consortium; https://www.nirsconsortium.org/).

Estimation of mid-day photosynthesis using PLSR, BR, ARDR, and LASSO presented lower *R*^2^ values (≈ 0.29–0.62) than for *V*_*c*,max_ and *J*_max_ (Fig. 3) since in situ photosynthetic measurements are likely more influenced by environment (Sanz-Sáez et al. 2017; Soba et al. 2020) than by leaf structure and biochemistry (Serbin et al. 2012; Ainsworth et al. 2014). Thus, a looser estimation was expected. Due to environmental variability, few reports have estimated mid-day photosynthesis. However, our PLSR estimation was better than the observations of Vitrack-Tamam et al. (2020) for cotton stomatal conductance (*R*^2^ = 0.23); this was likely due to the lower range spectral reflectance device used in their experiment (633–1659 nm). Similar estimations of net photosynthesis were accomplished using the scaled photochemical reflectance index and a FieldSpec Hi-Res Device (Kumari et al. 2012).

Regarding spectral wavelength specific coefficients for each estimation model for V_c,max_ and J_max_, the most frequent selection for the four models was the VIS waveband (Fig. 4) where chlorophyll and other pigments have strong absorption features (Peñuelas and Filella 1998). However, these models also used wavebands in the NIR and SWIR, similar to other studies (Hansen and Schjoerring 2003; Doughty et al. 2011; Serbin et al. 2012; Ainsworth et al. 2014; Yendrek et al. 2017). In addition, Rubisco has several relatively broad spectral absorption features in the NIR and SWIR (Elvidge 1990). These selections of spectral region combinations indicate that *V*_*c,*max_ and *J*_max_ spectral signatures are not simply a function of chlorophyll content, which suggests that more information is needed beyond the VIS–NIR wavebands to estimate such complex processes. The inclusion of a broader range of wavebands, due in part to less penalizations, is likely why the PLSR model outperformed BR, ARDR, and LASSO by more effectively capturing the broader spectral absorption features of Rubisco. For example, the *V*_*c*,max_ LASSO model only selected specific coefficients at 540, 680, 720, 2000, and 2250 nm (Fig. 4c), while the PLSR model had significant coefficient ranges between 400–450, 700–800, and 1750–1900 (Fig. 4c). Photosynthesis and LCC also presented the highest selection of spectral peaks in the VIS, followed by NIR; this has been extensively documented through both vegetation indices that estimate chlorophyll pigment content and also by the Photochemical Reflectance Index (PRI) that predicts photosynthetic efficiency through a zeaxanthin absorption feature (Gamon et al. 1997; Gitelson et al. 2005; Schlemmera et al. 2013).

We also present a more in-depth comparison of the four models. As shown in Fig. 3, the * R*^2^ of models do not present significant differences between each other, although we can see that the models used different numbers of coefficients to estimate each parameter (Fig. 4). This was reflected in the algorithm differences in each model approach to parsimony, the simple explanation of an occurrence involving the fewest entities, assumptions, or changes. This means that a fewer number of weight coefficients were used to estimate the different parameters (Vandekerckhove and Matzke 2015). In our study, all PLSR models (blue line in Fig. 4) used VIS, NIR, and SWIR wavelengths, but potentially over-fitted by an over-inclusion of predictor variables (Geladi et al. 1986; Wold et al. 2001). This contrasts to the BR (in green), ARDR (in red), and LASSO (in yellow) models (Fig. 4), which used more specific and limited spectra than restricted models that penalize the lesser coefficients (Neal 1996; Tibshirani 1996; Tipping 2001).

### Scaling up estimations of photosynthetic parameters for potential hyperspectral aerial or satellite applications

The second aim of this study was to simulate different sensors with more limited spectral coverage (VIS–NIR, NIR–SWIR, and SWIR), including the ESA Copernicus Sentinel-2 satellite13 bands. We found that estimation of *V*_*c*,max_ using three different sensor ranges (VIS–NIR–SWIR) with the four models performed (*R*^2^ = 0.50) surprisingly similar to the whole spectrum (Figs. 3 and 5). For *J*_max_, the highest estimation (*R*^2^ = 0.51) used NIR–SWIR and SWIR data in ARDR. This was quite similar to Meacham-Hensold et al. (2020) who used PLSR models and canopy-level spectra with three different spectral ranges (500–900, 500–1700, and 500–2400 nm) to achieve *V*_*c*,max_ estimations near *R*^2^ = 0.60 and *J*_max_ estimations around *R*^2^ = 0.40.

We also resampled FieldSpec data to cover the 12 spectral bands of the ESA Copernicus Sentinel-2 satellite; these were quite similar to spectral ranges selected by the coefficients used by the different models to estimate photosynthetic parameters. Concerning the different photosynthetic parameters, only *V*_*c*,max_ was estimated at more than *R*^2^ = 0.50. This could be related to the carboxylation process (*V*_*c*,max_) having several relatively broad spectral absorption features in NIR and SWIR centered at 1.5, 1.68, 1.74, 1.94, 2.05, 2.29 µm, etc. (Elvidge 1990), which are in close proximity to several Sentinel-2 wavelength bands (Table S2). Supplementary data (Table S3) and Serbin et al. (2012) showed that wavelengths (490, 610, 690, 710, 1680, 1940, 2200, 2400 nm) used to estimate *V*_*c*,max_ have some bands similar to Sentinel-2. Figure 5d also shows that the spectral regions used in PLSR models were similar to Sentinel bands (Yendrek et al. 2017). The limited success of single-leaf-level estimations of photosynthetic capacities using point-based spectral analysis (Serbin et al. 2015) found considerable promise in airborne and potential promise in space-borne imaging spectroscopy such as the NASA HyspIRI mission (Mariotto et al. 2013). In this regard, hyperspectral imagery through inversion of the Soil-Canopy Observation of Photosynthesis and Energy (SCOPE) model to estimate *V*_*c*,max_ also uses sensor resolutions available in airborne or even precision agriculture technologies (Camino et al. 2019). Recently, one plot-level study using sunlit vegetative reflectance pixels from a single visible near infra-red (VNIR; 400–900 nm) hyperspectral camera reported determination coefficients of *R*^2^ = 0.79 for *V*_*c*,max_ and *R*^2^ = 0.59 for *J*_max_ (Meacham-Hensold et al. 2020). Thus, our simulation analyses and other recent literature suggest that the wide range of variability in VIS, NIR, and SWIR sensors and the Sentinel-2 multispectral sensor (to a more limited extent) could be employed to estimate photosynthetic parameters (including *V*_*c*,max_ and *J*_max_) with advanced regression models. However, more research needs to be done in this area as one of the limitations of this work was that we measured leaf reflectance with a leaf clip, while UAV and satellites measure canopy reflectance that can be different from single leaf reflectance. For the future, we suggest to test if canopy reflectance measurements at different precision levels can predict leaf level photosynthetic measurements or even canopy-level photosynthesis as has been done with models such as PROSAIL (Berger et al. 2018).

## Conclusion and future directions

In this study, we estimated *V*_*c*,max_ and *J*_max_ using leaf spectral reflectance data and different advanced regression models with determination coefficients higher than *R*^2^ = 0.50–0.70. The combination of different species and environmental conditions (elevated [CO_2_], high temperature, and drought) increased phenotypic variation and improved model estimations where treatment effects were not significant. To achieve higher coefficients of determination and model performance, this research demonstrated that it is more important to have a wider range of phenotypic variation than a significant effect of a treatment or cultivar. We suggest that estimating photosynthetic capacity from reflectance spectra may be considered sufficiently robust to be useful for several different plant physiological applications, such as abiotic stress detection, improved characterization of photosynthesis process-based crop models, and a prescreening tool in breeding programs. We demonstrated that PLSR was the best model for predicting photosynthetic parameters in comparison to other advanced regression models (BR, ARDR and LASSO). However, new advance regression approaches that combine different regression models may be employed to increase phenotype estimation using this technology. Based on simulation of four limited spectral range sensors (VIS–NIR, NIR–SWIR and SWIR) using a leaf level spectrophotometer, we demonstrated that it is possible to estimate *V*_*c*,max_ with similar precision compared to using the whole VIS–NIR–SWIR spectrum. This research should encourage future studies using different imaging sensors (hyperspectral and multispectral) at different scales for estimating *V*_*c*,max_ and *J*_max_.

### *Author contribution statement*

MLB Experimentation, curation of the data, formal analysis, writing original draft. DS Experimentation, review and editing. TS Experimentation, review and editing. JL Experimentation, review and editing. IA Resource managing, review and editing. JLA Resource managing, review and editing. GBR Experimentation, review and editing. SAP Experimentation, review and editing, resource managing. SCK Conceptualization, data curation, resource managing, formal analysis, supervision, writing original draft. ASS Conceptualization, experimentation, data curation, resource managing, formal analysis, supervision, project administration, writing original draft.

## Supplementary Information

Below is the link to the electronic supplementary material.

Supplementary file1 (DOCX 1179 KB)

## Data Availability

The datasets generated during and/or analyzed during the current study are available from the corresponding author on reasonable request.

## Abbreviations

ARDR: Automatic relevance determination regression model
BR: Bayesian ridge model
*J*_max_: Maximum electron transport rate supporting RuBP regeneration
LASSO: Least absolute shrinkage and selection operator model
NIR: Near-infrared spectral reflectance
PLSR: Partial least squares regression model
RuBP: Ribulose 1,5-bisphosphate
SWIR: Shortwave infrared spectral reflectance
*V*_c,max_: Maximum rate of rubisco-catalyzed carboxylation
VIS: Visible spectral reflectance
