# Supplementary material for: Estimating peanut and soybean photosynthetic traits using leaf spectral reflectance and advance regression models
Source: Planta. 2022 Mar 24;255(4):93. doi: 10.1007/s00425-022-03867-6 (PMC8948130; doi:10.1007/s00425-022-03867-6)
Supplement: Supplementary file 1 — Supplementary file1 (DOCX 1179 KB) [file 425_2022_3867_MOESM1_ESM.docx]

Supplementary Figure and Tables

**Fig. S1 (a**) Mean, ± standard deviation, and minimum and maximum leaf reflectance for soybean. (**b**) Mean, ± standard deviation, and minimum and maximum leaf reflectance for peanut.


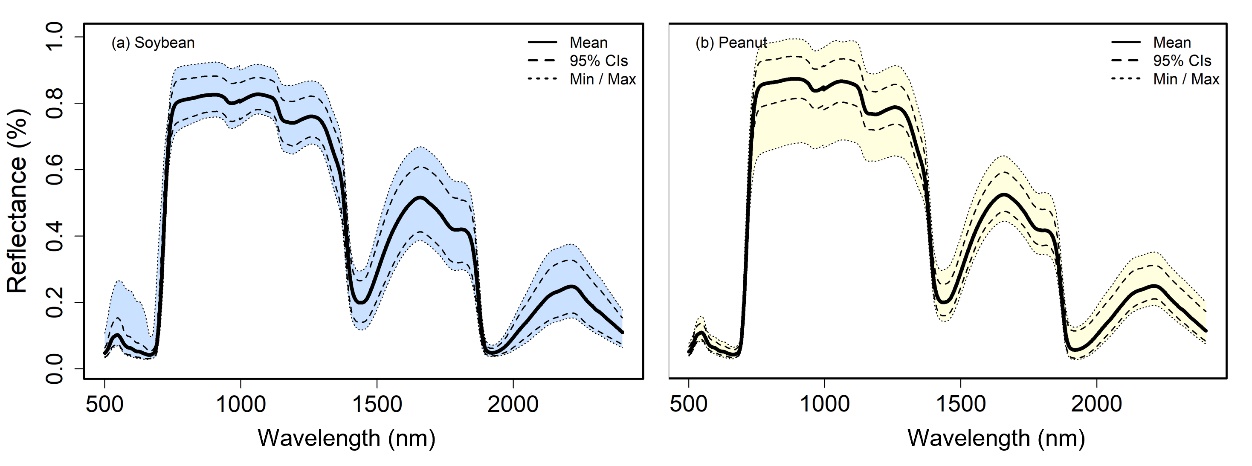



a)



b)

c)





# Fig. S2 Box-plot of midday photosynthesis (µmol m^-2^s^-1^), leaf chlorophyll content (LCC, arbitrary units), maximum rate of rubisco catalyzed carboxylation (V_c,max_, µmol m^-2^s^-1^), and leaf temperature (°C) per each treatment. (a) Experiment 1: two varieties of soybean grown under 410 ppm and 610 ppm of [CO_2_]. (b) Experiment 2: four soybean varieties grown under control (20°C) and high (30°C) night temperature. (c) Experiment 3: six varieties of peanut grown under well-watered (WW, 80% SWC) and water-stress (WS, 30% SWC) conditions.

| **Models using Peanut** | **Mid-day photosynthesis** | | **Leaf chlorophyll content (LCC)** | | **Maximum rate of rubisco catalyzed carboxylation** **(V_cmax_)** | | **Maximum electron transport rate supporting RuBP regeneration (J_max_)** | |
| --- | --- | --- | --- | --- | --- | --- | --- | --- |
|  |  |  |  |  |  |  |  |  |
|  |  |  |  |  |  |  |  |  |
|  | **R^2^** | **RMSE** | **R^2^** | **RMSE** | **R^2^** | **RMSE** | **R^2^** | **RMSE** |
| **PLSR** | 0.36 | 5.08 | 0.52 | 3.42 | -- | -- | -- | -- |
| **BR** | 0.27 | 5.44 | -- | -- | -- | -- | -- | -- |
| **ARDR** | 0.19 | 5.73 | 0.51 | 3.43 | -- | -- | -- | -- |
| **LASSO** | 0.19 | 5.73 | 0.39 | 3.54 | -- | -- | -- | -- |
| **Models using Soybean** | **Mid-day photosynthesis** | | **Leaf chlorophyll content (LCC)** | | **Maximum rate of rubisco catalyzed carboxylation** **(V_cmax_)** | | **Maximum electron transport rate supporting RuBP regeneration (J_max_)** | |
|  |  |  |  |  |  |  |  |  |
|  |  |  |  |  |  |  |  |  |
|  | **R^2^** | **RMSE** | **R^2^** | **RMSE** | **R^2^** | **RMSE** | **R^2^** | **RMSE** |
| **PLSR** | 0.24 | 5.28 | -- | -- | 0.70 | 46.36 | 0.69 | 30.13 |
| **BR** | 0.21 | 5.38 | -- | -- | 0.64 | 50.16 | 0.61 | 33.61 |
| **ARDR** | 0.22 | 5.35 | -- | -- | 0.49 | 60.03 | 0.59 | 34.64 |
| **LASSO** | 0.22 | 5.36 | -- | -- | 0.63 | 51.10 | 0.60 | 33.83 |

# Table S1 Coefficient of determination (R^2^) and Root Mean Squared Error (RMSE) of mid-day photosynthesis (µmol m^-2^s^-1^), leaf chlorophyll content (arbitrary units), maximum rate of rubisco catalyzed carboxylation (V_c,max_, µmol m^-2^s^-1^), and maximum electron transport rate supporting RuBP regeneration (J_max_, µmol m^-2^s^-1^) of peanut and soybean separately based on leaf reflectance spectra VIS-NIR-SWIR (350-2500nm) through advance regression models: Partial Least Squares Regression (PLSR), Bayesian Ridge (BR), Automatic Relevance Determination Regression (ARDR), and Least Absolute Shrinkage and Selection Operator (Lasso). The symbol “--" means that estimation models did not yield results due to lack of phenotypic variation.

# Table S2 Bands and resolutions of ESA Copernicus Sentinel-2 satellite wavelengths that were simulated in this manuscript.

| **Band Number** | **Part of the spectrum** | **Spectral Resolution (nm)** | **Central wavelength (nm)** | **Band Width (nm)** |
| --- | --- | --- | --- | --- |
| B1 | Coastal Aerosol | 3 | 443 | 36 |
| B2 | Blue | 3 | 494 | 96 |
| B3 | Green | 3 | 560 | 45 |
| B4 | Red | 3 | 665 | 39 |
| B5 | VRE | 3 | 704 | 20 |
| B6 | VRE | 3 | 740 | 18 |
| B7 | VRE | 3 | 781 | 28 |
| B8 | NIR | 3 | 834 | 141 |
| B9 | Water Vapour | 3 | 944 | 27 |
| B10 | SWIR Cirrus | 8 | 1375 | 76 |
| B11 | SWIR | 8 | 1612 | 142 |
| B12 | SWIR | 8 | 2194 | 240 |

#

# Table S3 Coefficient of determination (R^2^) and Root Mean Squared Error (RMSE) of mid-day photosynthesis (µmol m^-2^s^-1^), Leaf chlorophyll content (arbitrary units), maximum rate of rubisco catalyzed carboxylation (V_c,max_, µmol m^-2^s^-1^), and maximum electron transport rate supporting RuBP regeneration (J_max_, µmol m^-2^s^-1^) of soybean based on leaf reflectance spectra at different ranges: VIS-NIR (350-1000nm), NIR-SWIR (1000-2500nm), SWIR (1400-2500nm) and ESA Copernicus Sentinel-2 satellite bands through advance regression models: Partial Least Squares Regression (PLSR), Bayesian Ridge (BR), the Automatic Relevance Determination Regression (ARDR) and Least Absolute Shrinkage and Selection Operator (Lasso). “--" The estimation models did not yield results.

| **Estimation on Soybean** | | | | | | | | |
| --- | --- | --- | --- | --- | --- | --- | --- | --- |
| **Mid-day photosynthesis** | | | | | | | | |
| n = 77 | 350 to 1000 nm | | 1000 to 2500 nm | | 1400 to 2500 nm | | Simulation of Sentinel-2 | |
| **Model** | **R^2^** | **RMSE** | **R^2^** | **RMSE** | **R^2^** | **RMSE** | **R^2^** | **RMSE** |
| **PLSR** | -- | -- | -- | -- | 0.26 | 5.32 | 0.29 | 5.12 |
| **BR** | -- | -- | -- | -- | -- | -- | 0.21 | 5.38 |
| **ARDR** | -- | -- | -- | -- | -- | -- | 0.18 | 5.48 |
| **LASSO** | -- | -- | -- | -- | -- | -- | 0.2 | 5.44 |
| **Leaf chlorophyll content** | | | | | | | | |
| n = 77 | 350 to 1000 nm | | 1000 to 2500 nm | | 1400 to 2500 nm | | Simulation of Sentinel-2 | |
| **Model** | **R^2^** | **RMSE** | **R^2^** | **RMSE** | **R^2^** | **RMSE** | **R^2^** | **RMSE** |
| **PLSR** | -- | -- | -- | -- | 0.14 | 6.02 | -- | -- |
| **BR** | -- | -- | -- | -- | -- | -- | -- | -- |
| **ARDR** | -- | -- | -- | -- | -- | -- | -- | -- |
| **LASSO** | -- | -- | -- | -- | -- | -- | -- | -- |
| **Maximum rate of rubisco catalyzed carboxylation** **(V_c,max_)** | | | | | | | | |
| n = 81 | 350 to 1000 nm | | 1000 to 2500 nm | | 1400 to 2500 nm | | Simulation of Sentinel-2 | |
| **Model** | **R^2^** | **RMSE** | **R^2^** | **RMSE** | **R^2^** | **RMSE** | **R^2^** | **RMSE** |
| **PLSR** | 0.49 | 60.23 | -- | -- | 0.37 | 69.33 | 0.45 | 62.24 |
| **BR** | 0.63 | 46.57 | 0.29 | 70.59 | 0.39 | 65.46 | 0.60 | 53.19 |
| **ARDR** | 0.54 | 56.85 | 0.47 | 61.16 | 0.48 | 60.40 | 0.39 | 65.50 |
| **LASSO** | 0.64 | 50.57 | 0.46 | 61.46 | 0.45 | 62.45 | 0.66 | 49.01 |
| **Maximum electron transport rate supporting RuBP regeneration (J_max_)** | | | | | | | | |
| n = 81 | 350 to 1000 nm | | 1000 to 2500 nm | | 1400 to 2500 nm | | Simulation of Sentinel-2 | |
| **Model** | **R^2^** | **RMSE** | **R^2^** | **RMSE** | **R^2^** | **RMSE** | **R^2^** | **RMSE** |
| **PLSR** | 0.42 | 41.03 | 0.35 | 43.39 | 0.4 | 41.64 | 0.32 | 44.25 |
| **BR** | 0.65 | 31.92 | 0.55 | 36.07 | 0.55 | 36.06 | 0.54 | 36.42 |
| **ARDR** | 0.58 | 34.93 | 0.56 | 35.5 | 0.61 | 33.47 | 0.51 | 37.69 |
| **LASSO** | 0.56 | 35.73 | 0.55 | 36.13 | 0.56 | 35.88 | 0.59 | 34.24 |
